# Physical and chemical descriptors for predicting interfacial thermal resistance

**DOI:** 10.1038/s41597-020-0373-2

**Published:** 2020-02-03

**Authors:** Yen-Ju Wu, Tianzhuo Zhan, Zhufeng Hou, Lei Fang, Yibin Xu

**Affiliations:** 1 Center for Materials research by Information Integration (CMI2), Research and Services Division of Materials Data and Integrated System (MaDIS), National Institute for Materials Science (NIMS), 1-1 Namiki, Tsukuba, Ibaraki 305-0044 Japan; 2 Waseda University, 3-4-1 Okubo, Shinjuku-ku, Tokyo 169-8555 Japan; 3 State Key Laboratory of Structural Chemistry, Fujian Institute of Research on the Structure of Matter, Chinese Academy of Sciences, Fuzhou, Fujian 350002 China

**Keywords:** Computational methods, Synthesis and processing

## Abstract

Heat transfer at interfaces plays a critical role in material design and device performance. Higher interfacial thermal resistances (ITRs) affect the device efficiency and increase the energy consumption. Conversely, higher ITRs can enhance the figure of merit of thermoelectric materials by achieving ultra-low thermal conductivity via nanostructuring. This study proposes a dataset of descriptors for predicting the ITRs. The dataset includes two parts: one part consists of ITRs data collected from 87 experimental papers and the other part consists of the descriptors of 289 materials, which can construct over 80,000 pair-material systems for ITRs prediction. The former part is composed of over 1300 data points of metal/nonmetal, nonmetal/nonmetal, and metal/metal interfaces. The latter part consists of physical and chemical properties that are highly correlated to the ITRs. The synthesis method of the materials and the thermal measurement technique are also recorded in the dataset for further analyses. These datasets can be applied not only to ITRs predictions but also to thermal-property predictions or heat transfer on various material systems.

## Abstract (summary)

**Table:** 

Measurement(s)	material entity • interface • Material Description
Technology Type(s)	machine learning • digital curation

Machine-accessible metadata file describing the reported data: 10.6084/m9.figshare.11618253

## Background & Summary

The interfacial thermal resistance (ITR) has become the dominant factor controlling the nano/micro device performance. A high thermal resistance at interfaces decreases heat dissipation and electron injection, resulting in lower efficiency and larger energy consumption. Conversely, thermal insulating thin films can be achieved via nanostructuring design and high-ITR material system selection. Commonly used prediction methods of ITR, the acoustic mismatch model (AMM) and the diffuse mismatch model (DMM), show large mismatches between experimental and predictive results with a low prediction performance of 60%^1,2^. This low predictive performance implies that there are additional properties that affect the ITR and need to be included.

Machine learning has become a potential powerful means to accelerate the development of interfaces for thermal management from the hundreds of thousands of possible candidates. Yang *et al*. predicted the ITR between graphene and hexagonal boron nitride for high-performance thermal interface materials using different machine-learning algorithms, in which the and deep neural networks showed the best predictive results^3^. Sosso *et al*. efficiently built interatomic potentials for the thermal properties of amorphous materials using machine learning while retaining the accuracy of first-principle calculations^4^. Gaultois *et al*. demonstrated promising new thermoelectric compounds via the pre-screening of 25,000 known materials and then confirmed their thermoelectric properties experimentally^5^. In a previous study, we proposed electrically conductive thermally insulating Bi/Si composite thin films^6^, which was a high-ITR material system selected by a machine-learning prediction model^1^. This ITR prediction model showed a higher predictive performance (93%) than AMM and DMM models. The nanostructure of the Bi/Si thin films was optimized via combinatorial sputtering, and the high surface/volume ratio of the Bi particles in the Si matrix and high ITR of the Bi/Si interfaces contributed to the ultra-low thermal conductivity (0.16 W/mK) of the material, which is as low as that of polymers^6^. Both the predictive performance and the experimental results proved the potential practical use of ITR prediction models for interface designs for thermal management.

**Fig. 1:**
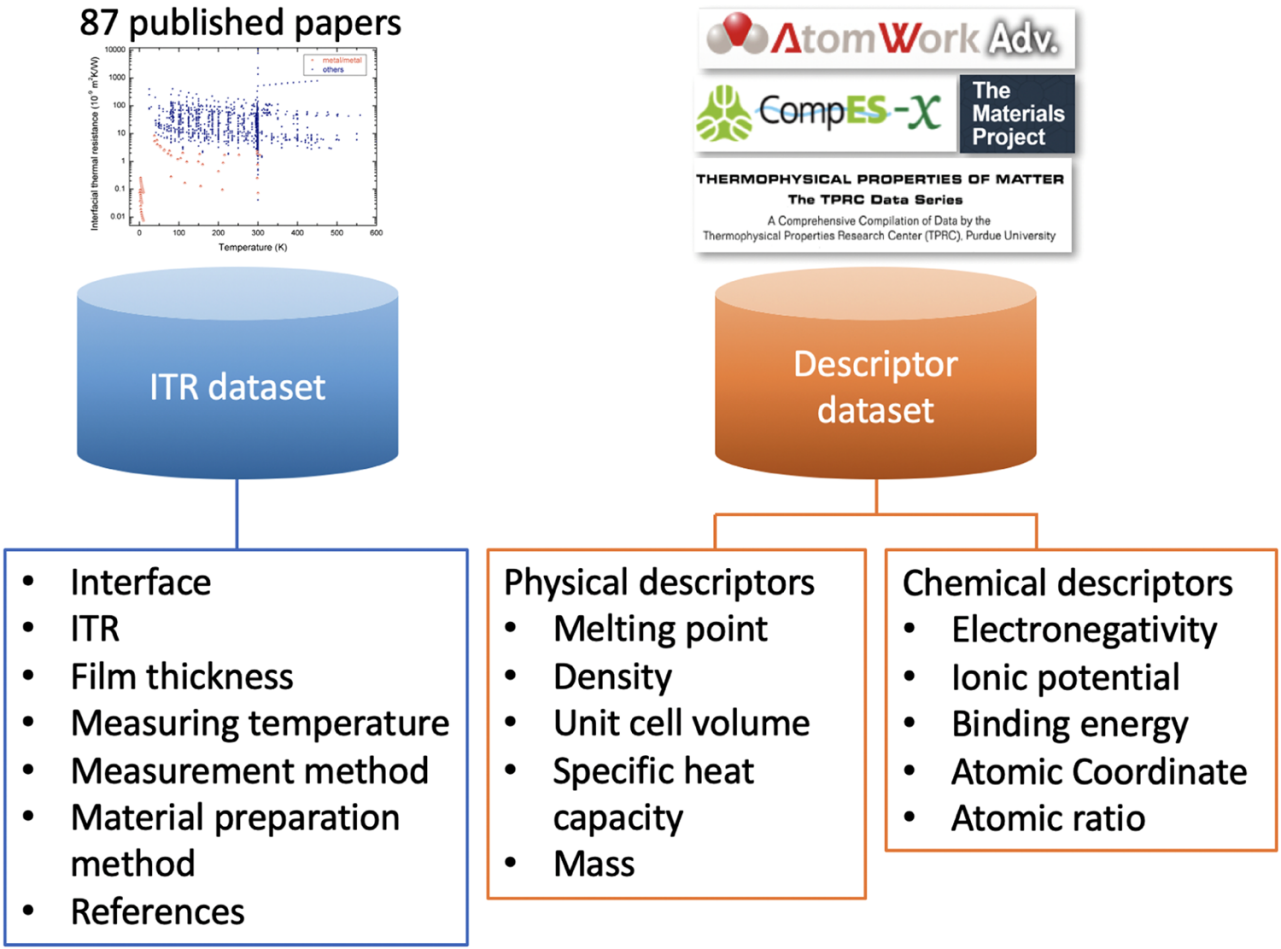
A schematic overview of the ITR and descriptor datasets. The ITR dataset includes experimental data collected from 87 papers, the experimental conditions, and their reference details. The descriptor datasets are composed of the physical and chemical descriptors of different materials that can be used for data training and/or prediction via machine learning.

The above ITR prediction model was trained using experimental ITR data from 87 published papers including not only thermal physical descriptors, such as the unit cell volume and the density used in AMM and DMM, but also chemical descriptors (e.g., binding energy, electronegativity, and ion potential) and process descriptors (e.g., film thicknesses and interlayers). The collected descriptors have a high data-consistency between the references and a high data-availability, and the details of the descriptor selection can be found in our previous papers^1,2^. Here, we present the details of the two datasets we used for the ITR model training and prediction, as shown in Fig. 1; one is the collected ITR dataset and the other is the descriptor dataset of various materials. The former dataset shows the ITR values of various interfaces including the temperature, synthesis method, thermal measurement method, sample pretreatment, and its original references. This dataset can be further categorized by the material systems based on the analysis purpose, for example, comparing the ITR range between metal/metal and metal/nonmetal interfaces. The latter dataset shows the physical, chemical, and process descriptors of 298 different materials, which are single element or binary compounds. These materials can be used to construct over 80,000 pair-material systems (e.g., Bi/Si) for ITR prediction.

The datasets have multiple uses: (1) the ITR prediction model can be constructed according to the ITR and descriptor datasets for interface designs of thermoelectric materials or highly efficient electronic devices, (2) the descriptor dataset of the 298 materials can be re-used for other predictions with different targets (e.g., thermal conductivity), and (3) the correlation between the target and descriptors or the similarities between materials can be visualized via linear/nonlinear analyses. The collected datasets can therefore accelerate the development of material designs to improve interfacial thermal management.

## Methods

The ITR data were collected from the experimental data in 87 published papers^1,7–92^; some of them were extracted from plots via WebPlotDigitizer (https://automeris.io/WebPlotDigitizer)^93^. The interfacial thermal resistance (10^−9^ m^2^ K/W), thermal boundary conductance (MW/m^2^K), material system of the interfaces in chemical formula (e.g., Bi/Si), temperature (K), and film thickness were compiled. Moreover, the associated preparation methods for the materials, such as sputtering and evaporation, measurement methods of ITR, pretreatment of substrates, and other details concerning the interfaces, were collected if they were mentioned in the references.

The descriptor dataset includes the specific heat capacity, melting point, density, unit cell volume, electronegativity (EN), ionic potential (IP), atomic ratio (R), mass, atomic coordinate (AC), and binding energy (E_b_) of 298 materials. The atomic ratios of the compounds for the first and second elements were defined as R_1_ and R_2_, respectively. For example, for SiO_2_, R_1_ and R_2_ are 1 and 2, respectively. AC represents the atomic coordinates defined in the periodic table, with the group as the x-coordinate and the period as the y-coordinate, e.g., (AC_i_x, AC_i_y), where i represents the order of the elements of the compound. For example, for GaN, the coordinates of (AC_1_x, AC_1_y) and (AC_2_x, AC_2_y) are (13, 4) and (15, 2), respectively.

The specific heat capacity was collected from the TPRC data series^94^; the melting point, density, and unit cell volume were collected from AtomWork-Adv by the National Institute for materials Science (NIMS) (https://atomwork-adv.nims.go.jp/)^95^; EA, IP, and the mass were collected from the periodic table via the Pauling scale and National Institute of Standards and Technology (NIST)^96,97^; and E_b_ was calculated from the total energy of relaxed crystal structure of compound, which was collected in the Computational Electronic Structure Database (CompES-X)^98^. CompES-X is a database of electronic structures predicted by the first-principle calculations for mono-element and multi-element crystalline inorganic compounds based on experimental data of crystal structures. The total energies of constituent atoms can be found in the atom_energy_vasp sheet at 10.5281/zenodo.3564173 ^99^, in which the isolated atom was simulated by putting one atom in a cubic supercell with a length of 15 Å and was calculated using the same computational method as the one for compounds in CompES-X. For example, the binding energy of TiO_2_, E_b_[TiO_2_], is calculated according to Eq. (1).

1 $${{\rm{E}}}_{{\rm{b}}}\left[{{\rm{TiO}}}_{2}\right]={{\rm{E}}}_{{\rm{tot}}}\left[{{\rm{TiO}}}_{2({\rm{bulk}})}\right]-{{\rm{E}}}_{{\rm{tot}}}\left[{{\rm{Ti}}}_{({\rm{atom}})}\right]-2{{\rm{E}}}_{{\rm{tot}}}\left[{{\rm{O}}}_{({\rm{atom}})}\right],$$

where E_tot_[TiO_2(bulk)_] is the total energy of bulk TiO_2_ and E_tot_[Ti_(atom)_] and E_tot_[O_(atom)_] are the total energies of isolated Ti and O atoms, respectively.

## Data Records

### ITR dataset

The ITR dataset contains 1318 data (id) composed of 457 interface (interface id) samples and 54 materials, including metals, insulators, and semiconductors. The 457 interfaces are defined by their films, interlayers, substrate materials, and experimental conditions. Take the Au/SiO_2_/Si interfaces in Table 1 for example: all the Au/SiO_2_/Si data from ids 1 to 5 used the same sample measured at different temperatures from 100 K to 296 K; therefore, the interface ids are all defined as being the same. Each interface is depicted by its chemical formula or name separated by a slash, for example, Al/Si, as shown in Table 1. To input the data for machine learning, there are six materials that use abbreviations in the “Film 1” and “Film 2” columns; C for diamond, gp-C for graphene, g-C for graphite, a-SiO_2_ for glass, SiO_2_ for quartz, and Al_2_O_3_ for sapphire. Note that most of the Film 2 entries are substrates based directly on the commonly used measurement methods, such as time domain thermoreflectance (TDTR) or frequency domain thermoreflectance (FDTR)^55,83^. For some of the others, the Film 2 entry is not the substrate itself and the ITR values at the Film 2/substrate have been extracted or eliminated from the total resistance. Accordingly, the materials in the “Film 2” and “substrate details” columns of some interfaces are not consistent, such as those of Au/TiO_2_ and Au/a-SiO_2_ in Table 1. The interlayer column reflects whether an interlayer is present between the materials (Film 1/Film 2) at the interface; this value is either 1 or 0 (the former if an interlayer is present, and the latter if interlayers are absent) For example, the interlayers of Cr/Si and Cr/a-Si/Si in Table 1 are defined as 0 and 1, respectively. The interlayer includes the adhesion layer, a naturally or thermally formed oxidation layer (e.g., Au/SiO_2_/Si in Table 1)^55^, and the surface plasma treatment (e.g., the Bi/H-diamond in Table 1), which forms interlayers or a mixed region between the materials instead of a clear interface. The information concerning the experimental and interfacial conditions can be found in the substrate pretreatment columns, and other interfacial properties can be found in the file of “ITR dataset” at 10.5281/zenodo.3564173^99^. Further details can be found in the “ITR Reference” sheet using the reference-tracking id (id-R).

**Table 1:** The ITR dataset collected from the 87 papers. There are 11 data points given as examples including the interface id, interface, interlayer (1: exists, 0: absent), ITR, temperature, measurement method, materials for the film and substrate, the preparation method, film thickness, substrate details, and reference id. The columns showing the substrate details, substrate pretreatment, and interfacial properties are not listed here; this information can be found at 10.5281/zenodo.3564173^99^. The reference id (id-R) corresponds to the sheet of ITR references at 10.5281/zenodo.3564173^99^.

id	interface id	Interface	interlayer	ITR (10^−9^ m^2^K/W)	Measuring temperature (K)	Measurement method	Film 1	Film 1 preparation method	Film 1 thickness (nm)	substrate (Film2)	Substrate details	Reference (id-R)
1	1	Au/SiO_2_/Si	1	26.3157895	100	TDTR	Au	e-beam evaporation	80	Si	Boron-doped Si (100)	^1^
1	1	Au/SiO_2_/Si	1	26.3157895	100	TDTR	Au	e-beam evaporation	80	Si	Boron-doped Si (100)	^1^
2	1	Au/SiO_2_/Si	1	24.3902439	150	TDTR	Au	e-beam evaporation	80	Si	Boron-doped Si (100)	^1^
3	1	Au/SiO_2_/Si	1	25.6410256	200	TDTR	Au	e-beam evaporation	80	Si	Boron-doped Si (100)	^1^
4	1	Au/SiO_2_/Si	1	22.7272727	250	TDTR	Au	e-beam evaporation	80	Si	Boron-doped Si (100)	^1^
5	1	Au/SiO_2_/Si	1	21.2765957	296	TDTR	Au	e-beam evaporation	80	Si	Boron-doped Si (100)	^1^
119	17	Al/Si	0	5.18134715	298	TDTR	Al	evaporation	80	Si	Phosphorus-doped Si (100)	^4^
153	30	Bi/H-diamond	1	256.410256	80	TDTR	Bi	thermal evaporation	100	C	Hydrogen-terminated Diamond	^6^
230	60	Cr/Si	0	8.84955752	298	TDTR	Cr	Sputter deposition	50	Si	Si	^12^
231	61	Cr/a-Si/Si	1	5.61797753	298	TDTR	Cr	Sputter deposition	50	Si	Si	^12^
232	62	Au/TiO_2_	0	25	298	TDTR	Au	magnetron sputtering	50	TiO_2_	Si	^10^
479	154	Au/a-SiO2	0	4.5045045	298	2 ω	Au	thermal evaporation	100	a-SiO_2_	Si	^26^

### Descriptor dataset

The descriptor dataset is composed of the physical and chemical descriptors of 298 materials. The former includes the specific heat capacity, melting point, density, unit cell volume, and mass, and the latter includes the electronegativity (EN), IP, atomic ratio (R), atomic coordinate (AC), and binding energy (E_b_). The materials are single element or binary compounds and are assigned a material id (id-M), as shown in Table 2. The units for the specific heat capacity, melting point, density, unit cell volume, mass, IP, and E_b_ are J/gK, K, g/cm^3^, 10^−29^ m^3^/formula unit (f.u.), u, eV, and eV/f.u., respectively; while the other quantities are dimensionless.

**Table 2:** The descriptor dataset for 12 different materials is shown as an example. The material id (id-M), material, formula, specific heat capacity, melting point, density, volume per formula unit (f.u.), atomic ratio (R), mass, atomic coordinate (AC), electronegativity (EN), ionic potential (IP), and binding energy (Eb) can be found in the dataset.

id-M	Material	Formula	Specific heat capacity (J/gK)	Melting point (K)	Density (g/cm^3^)	Volume per f.u. (10^−29^ m^3^/f.u.)	R1	R2	Mass (u)	AC1x	AC1y	AC2x	AC2y	ENc	ENa	IPc	IPa	Eb(eV/f.u.)
1	Silicon	Si	0.71	1687	2.33	2	1	1	28.09	14	3	14	3	1.9	1.9	8.15	8.15	−4.62
2	Germanium	Ge	0.31	1211	5.34	2.26	1	1	72.64	14	4	14	4	2.01	2.01	7.9	7.9	−3.86
3	Glass	a-SiO_2_	0.75	1873	2.2	4.53	1	2	60.08	14	3	16	2	1.9	3.44	8.15	13.62	−19.65
4	Gold	Au	0.13	1337	19.3	1.7	1	1	196.97	11	6	11	6	2.54	2.54	9.23	9.23	−3.81
5	Aluminium	Al	0.9	934	2.7	1.65	1	1	26.98	13	3	13	3	1.61	1.61	5.99	5.99	−3.39
6	Lead	Pb	0.13	600	11.4	3.03	1	1	207.2	14	6	14	6	2.33	2.33	7.42	7.42	−2.03
7	Bismuth	Bi	0.12	545	9.8	3.52	1	1	208.98	15	6	15	6	2.02	2.02	7.29	7.29	−2.18
8	Titanium	Ti	0.52	1953	4.5	1.76	1	1	47.87	4	4	4	4	1.54	1.54	6.82	6.82	−4.85
9	Chromium	Cr	0.45	2118	7.2	1.2	1	1	52	6	4	6	4	1.66	1.66	6.77	6.77	−4.09
10	Titanium nitride	TiN	0.6	3200	5.5	1.91	1	1	61.87	4	4	15	2	1.54	3.04	6.82	14.53	−13.58
11	Magnesium oxide	MgO	0.9	3125	3.5	1.87	1	1	40.3	2	3	16	2	1.31	3.44	7.65	13.62	−10.25
12	Sapphire	Al_2_O_3_	0.78	2300	3.99	4.26	2	3	101.96	13	3	16	2	1.61	3.44	5.99	13.62	−31.79

## Technical Validation

**Fig. 2:**
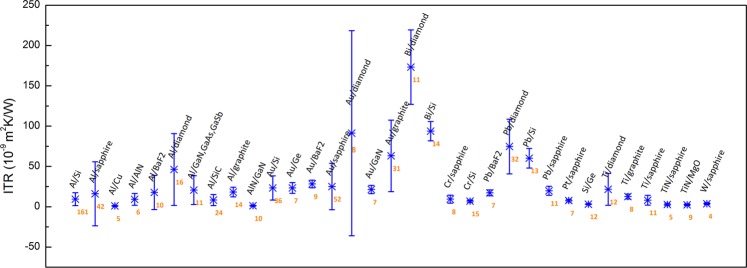
An ITR statistical plot of the ITR dataset. The data number of each material system is depicted in orange.

In this section, we present the statistical analyses and experimental variations of the ITR dataset and use the data selection of the ITR prediction as an example. First, the experimental data distribution is demonstrated in Fig. 2. Most of the material systems show small standard deviations, and Al/Si has the largest amount of data at 106 points. Al and Au have high percentages as film materials in the dataset because these materials are commonly used as heat transducer layers to absorb laser heat via TDTR and FDTR measurements^55,83^. Of the material systems, Au/Si has the largest standard deviation, which can be attributed to its unique experimental conditions including heavy ion bombardment or plasma treatment^62,74^. For machine learning, too sparse data sometimes can lead to a big challenge on the data training. Except for data with special treatments, the heat transport modes and main carriers of the metal/metal interface or two-dimensional (2D) materials are different compared to the metal/nonmetal interface materials. Therefore, the material systems composed of 2D materials, such as graphene and metal/metal, or materials that have no exact composition ratio, were removed from the dataset for the ITR prediction model. However, the data selection criteria change depending on the purpose. If one focuses on thermal transport at metal/diamond, Si, or sapphire interfaces, then surface treatments such as H-plasma or bombardment would be helpful for broader considerations and comparisons.

**Fig. 3:**
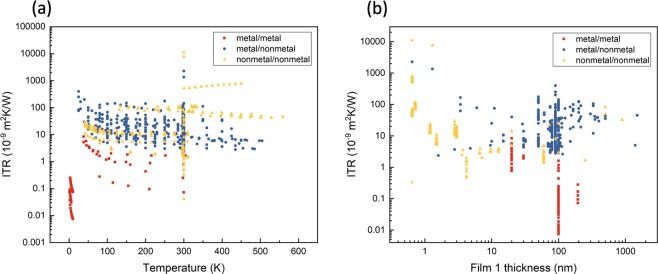
The ITR data distribution without an interlayer. The ITR data distribution versus the temperature and the film 1 thickness are shown in (**a**,**b**), respectively. The data include three types of material systems: metal/metal in red, metal/nonmetal in blue, and nonmetal/nonmetal in yellow.

**Fig. 4:**
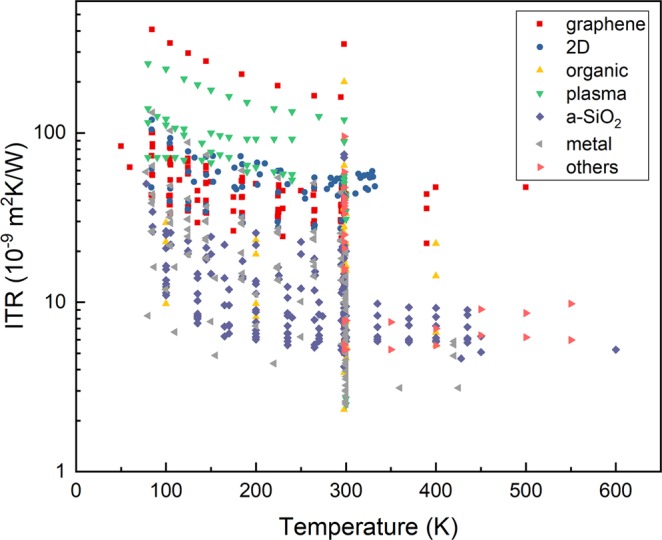
The ITR data distribution with interlayers versus the temperature. The interlayer materials are categorized into seven groups: graphene (red), other 2D materials (blue), organic materials (yellow), surface plasma treatment (green), amorphous SiO_2_ (a-SiO_2_) (purple), metal (gray), and others (pink).

To further verify the ITR data for other specific thermal analysis, the ITR data distribution with and without an interlayer are shown in Figs. 3 and 4, respectively. The ITR data without an interlayer are categorized into three groups of metal/metal, metal/nonmetal, and nonmetal/nonmetal in Fig. 3. ITR decreases for the most part with increasing temperature in Fig. 3(a), and the ITR values of metal/metal are two to four orders lower than those of metal/nonmetal and nonmetal/nonmetal. In Fig. 3(b), a thickness dependence is not obvious for the different groups and a thickness near 100 nm is most commonly used due to laser absorption depth considerations. The ITR data organized into seven different interlayer groups versus the temperature are shown in Fig. 4. Even though the ITR values depend on the different material systems, the interlayer materials affect the ITR values as well: the 2D material group (including graphene) has relatively higher ITR values while the metal group tends to have lower ITR values.

## Usage Notes

A description of the two datasets, the ITR and descriptor datasets, as well as the calculated total energy of isolated atoms via first-principle calculations (atom_energy_vasp), are provided. Further, the training data for the ITR machine-learning model are furnished under the file name “training dataset for ITR prediction” and can be directly used as training data for ITR predictions. Accordingly, the archive contains of four files with their depicted content, units, and sheets is shown in Online-only Table 1. This table can assist in searching for the data locations for broad thermal management; in addition, each ITR data point can be tracked via its reference id (id-R) in the “ITR References” sheet for further information. All the datasets can be found in 10.5281/zenodo.3564173 ^99^.

The datasets can be applied for flexible research purposes as mentioned above in the section of Background & Summary, here we take predicting ITR as an example. The construction steps are simply described in the following:

1. The target of ITR and the descriptors which are related to ITR should be input for training the machine learning model. Taking the interface of Al/Si as one example, the experimental ITR at different temperature (if available in papers) and the chemical, physical descriptors of both Al and Si should be collected.
2. The file “training dataset for ITR prediction” in 10.5281/zenodo.3564173 ^99^, which includes the experimental ITR data and materials’ descriptors, can be used as training dataset directly.
3. And then training the model by tuning the hyper parameters via cross validation. The machine learning model is usually evaluated by the mean square error and R^2^.
4. Once you achieve good predictive performance, you can input various material systems such as Si/Ge with specific temperature, film thickness and their properties for prediction.
5. The potential candidates from the prediction could be further analyzed via experiments or simulation.

The details of descriptor selection, algorithm selection, and prediction analysis for the ITR machine-learning model and its applications can be found in our previous studies^1,6^. Before applying the training dataset, “training dataset for ITR prediction,” we provided, there are some prerequisite restrictions you should consider corresponding to your research: (1) The training data excluded the metal/metal interface, two-dimensional (2D) materials, materials that have no exact composition ratio, and the interfaces with special treatments such as heavy ion bombardment from the original file “ITR dataset”. (2) The chemical and physical descriptors were collected from data platform (AtomWork-Adv)^95^ or handbooks (TPRC data series)^94^ due to the limited information from the original papers. Therefore, there may be some mismatch between the materials and their descriptors, such as density and unit cell volume. (3) The data distribution is different corresponding to various material system or samples. For example, the data number of Al/Si is much more than other material systems. Besides, the ITR dataset contains 1318 data composed of only 457 interface samples because some samples have many ITR data points corresponding to different temperatures. For the prediction purpose, the temperature could be calibrated to prevent the data distortion.

## Online-only Table

**Online-only Table 1:** Overview of the archive content. A demonstration of the data is provided for each file.

File	Sheet	Content	Unit
ITR dataset.xlsx	ITR	id	
		interface id	
		interface	
		interlayer	
		Thermal boundary conductance	MW/m^2^K
		ITR	10^−9^ m^2^K/W
		Temperature	K
		Measuring method	
		Film	
		Film preparation method	
		Film thickness	nm
		Substrate	
		Substrate details	
		Substrate pretreatment	
		Interfacial properties	
		References (id-R)	
	ITR References	Id-R	
		Title	
		Authors	
		Journal	
		DOI	
Descriptor dataset.xlsx	Descriptor dataset	Id-M	
		Material	
		formula	
		Specific heat capacity	J/gK
		Melting point	K
		Density	g/cm^3^
		Volume per formula unit (f.u.)	10^−29^ m^3^/f.u.
		R (atomic ratio)	
		mass	u
		Atomic coordinate (AC)	
		Electronegativity (EN)	
		Ionic potential (IP)	
		Binding energy (Eb)	eV/f.u.
atom_energy_vasp.xlsx	atom_energy_vasp	atomic number	
		symbol	
		PAW potential name	
		Total energy of an isolated atom	eV/atom
training dataset for ITR prediction.xlsx	training dataset	The arranged dataset for data training.	
		The details can be found in README sheet.	
	README		
