# Author Correction: Physical and chemical descriptors for predicting interfacial thermal resistance

**DOI:** 10.1038/s41597-020-0405-y

**Published:** 2020-02-17

**Authors:** Yen-Ju Wu, Tianzhuo Zhan, Zhufeng Hou, Lei Fang, Yibin Xu

**Affiliations:** 1 Center for Materials research by Information Integration (CMI2), Research and Services Division of Materials Data and Integrated System (MaDIS), National Institute for Materials Science (NIMS), 1-1 Namiki, Tsukuba, Ibaraki 305-0044 Japan; 2 Waseda University, 3-4-1 Okubo, Shinjuku-ku, Tokyo 169-8555 Japan; 3 State Key Laboratory of Structural Chemistry, Fujian Institute of Research on the Structure of Matter, Chinese Academy of Sciences, Fuzhou, Fujian 350002 China

Correction to: *Scientific Data* 10.1038/s41597-020-0373-2, published online 03 February 2020

Following publication of this Data Descriptor, it was found that references in the “Reference (id-R)” column of Table 1 that were intended to correspond to references in the ITR dataset.xlsx file of the accompanying dataset were incorrectly displayed as bibliographic references. Table 1 has been updated to make this distinction clear in both the HTML and PDF versions.

